# Value of the 21-gene expression assay in predicting locoregional recurrence rates in estrogen receptor-positive breast cancer: a systematic review and network meta-analysis

**DOI:** 10.1007/s10549-022-06580-w

**Published:** 2022-04-15

**Authors:** Matthew G. Davey, Eoin F. Cleere, John P. O’Donnell, Sara Gaisor, Aoife J. Lowery, Michael J. Kerin

**Affiliations:** Department of Surgery, The Lambe Institute for Translational Research, National University of Ireland, Galway, Galway, H91 YR71 Republic of Ireland

**Keywords:** Breast cancer, Locoregional recurrence, Cancer genomics, Personalized medicine

## Abstract

**Purpose:**

The Oncotype DX© 21-gene Recurrence Score (RS) estimates the risk of distant disease recurrence in early-stage estrogen receptor-positive, human epidermal growth factor receptor-2-negative (ER+/HER2− ) breast cancer. Using RS to estimate risk of locoregional recurrence (LRR) is less conclusive. We aimed to perform network meta-analysis (NMA) evaluating the RS in estimating LRR in ER+/HER2− breast cancer.

**Methods:**

A NMA was performed according to PRISMA-NMA guidelines. Analysis was performed using R packages and Shiny.

**Results:**

16 studies with 21,037 patients were included (mean age: 55.1 years (range: 22–96)). The mean RS was 17.1 and mean follow-up was 66.4 months. Using traditional RS cut-offs, 49.7% of patients had RS < 18 (3944/7935), 33.8% had RS 18–30 (2680/7935), and 16.5% had RS > 30 (1311/7935). Patients with RS 18–30 (risk ratio (RR): 1.76, 95% confidence interval (CI): 1.32–2.37) and RS > 30 (RR: 3.45, 95% CI: 2.63–4.53) were significantly more likely to experience LRR than those with RS < 18. Using TAILORx cut-offs, 16.2% of patients had RS < 11 (1974/12,208), 65.8% had RS 11–25 (8036/12,208), and 18.0% with RS > 30 (2198/12,208). LRR rates were similar for patients with RS 11–25 (RR: 1.120, 95% CI: 0.520–2.410); however, those with RS > 25 had an increased risk of LRR (RR: 2.490, 95% CI: 0.680–9.390) compared to those with RS < 11. There was a stepwise increase in LRR rates when applying traditional and TAILORx cut-offs (both *P* < 0.050).

**Conclusion:**

RS testing accurately estimates LRR risk for patients being treated for early-stage ER+/HER2− breast cancer. Future prospective, randomized studies may validate the predictive value of RS in estimating LRR.

**Supplementary Information:**

The online version contains supplementary material available at 10.1007/s10549-022-06580-w.

## Introduction

Breast cancer is the most frequently diagnosed malignancy in women, with a lifetime risk of 12.4% in the western world [1]. While surgical resection through Halsted’s radical mastectomy was once considered the cornerstone of breast cancer management, novel therapeutic strategies and a more targeted approach to surgery have facilitated the improved oncological and survival outcomes, while minimizing treatment-related morbidity [2]. These timely changes to breast cancer care coincide with our heightened appreciation for the cellular, biomolecular, and genomic properties responsible for driving oncogenesis [3, 4]. Moreover, these advances have facilitated the development and incorporation of multigene expression assays into the clinical paradigm for breast cancer management to personalize treatment strategies [5–7].

Within the setting of early-stage estrogen receptor-positive, human epidermal growth factor receptor-2-negative (ER+/HER2− ) breast cancer, the Oncotype DX© Recurrence Score (RS) 21-gene expression assay (commercially available at Genomic Health Inc., Redwood City, California, United States) is used to predict distant disease recurrence following treatment with curative intent and to estimate prognosis [8, 9]. This assay uses reverse transcriptase polymerase chain reaction to determine expression levels of 16 cancer related and 5 control genes from the resected tumor specimen, which are then incorporated into an algorithm to provide the clinician with a RS and applied clinically to guide chemoendocrine prescription for early-stage ER+/HER2− disease [5]. The rapid translation of the 21-gene expression assay into the clinical management of breast cancer has successfully de-escalated the prescription of systemic chemotherapy in those with low-to-intermediate risk molecular profiles of experiencing recurrence [8, 10].

While the 21-gene expression assay is useful in gauging the potential benefit of prescribing cytotoxic chemotherapy in high-risk cases, its utility in estimating LRR risk is less apparent: Tumor blocks retrieved from the NSABP-B14 and B20 trials were evaluated with the sole purpose of establishing the risk of distant disease recurrence in a subset of ER+/HER2− lymph node-negative (LN-) breast cancer patients, before being combined to develop the RS signature [5]. The predictive value of using RS to estimate locoregional recurrence (LRR) in early-stage ER+/HER2− disease is uncertain. The prevention of LRR is crucial in breast cancer, as reducing LRR translates directly into reduced breast cancer-related death [11]. Despite this, estimating the risk of LRR in early-stage ER+/HER2− often proves challenging to the clinician. Accordingly, the aim of the current systematic review and network meta-analysis (NMA) was to evaluate the clinical utility of the 21-gene expression assay in estimating LRR in early-stage ER+/HER2− breast cancer.

## Methods

A systematic review was conducted in accordance to the ‘Preferred Reporting Items for Systematic Reviews and Meta-Analyses’ (or PRISMA) extension statement for reporting of systematic reviews incorporating network meta-analyses of healthcare interventions [12]. Local institutional ethical approval was not sought as all data used in this analysis were obtained from a previously published resource.

### Study eligibility

All published studies with full-text manuscripts comparing LRR rates per RS category following treatment with curative intent for early-stage ER+/HER2− breast cancers were included. Randomized controlled trials and observational studies of a prospective and retrospective design were included. Included studies were expected to report on the primary outcome of interest. All studies failing to fulfill the pre-determined inclusion criteria were excluded. Conference abstracts, case reports, case series with less than 5 patients, editorial articles, opinion pieces, and review articles were excluded. Our rationale for omitting such articles was due to the paucity of crude data available in such forms for inclusion in meta-analysis. Studies not published in the English language were excluded. Included studies were not restricted by year of publication.

### Population, intervention, comparison, and outcome (PICO)

Using the PICO framework [13], the aspects the authors wished to address were as follows:

Population–Patients who had previously been diagnosed with an invasive ER+/HER2− breast cancer aged 18 years or older who had undergone RS testing on their resected specimen from time of diagnosis.

Intervention/Exposure–Any patient who subsequently developed LRR during follow-up (which includes those who have developed LRR with or without distant disease recurrence).

Comparison/Control–Any patient free of LRR during follow-up.

Outcomes–The primary outcome of interest was as follows:

- LRR rates for each RS category (using traditional [5] and TAILORx [8] cut-offs).

The secondary outcomes of interest included:

- Clinicopathological, surgical, and adjuvant treatment data for all patients.

### Search strategy

A formal systematic search of the PubMed, Embase, and Scopus electronic databases was performed for titles studies relevant to this research question. This search was performed by two independent reviewers (MGD & EFC), using a pre-determined search strategy that was designed by the senior author (MJK). This search included the search terms: [(21-gene assay) OR (oncotype)] AND (locoregional recurrence) linked using the Boolean operator ‘AND.’ Manual cross-referencing of reference lists from previous studies was undertaken.

Manual removal of duplicate studies was performed before all titles were screened. Thereafter, studies considered to be appropriate had their abstracts and/or full text reviewed. Retrieved studies were reviewed to ensure inclusion criteria were met for the primary outcome, with discordances in opinion arbitrated through consultation with a third author (JPOD). Data extraction was also performed by two independent reviewers (MGD & EFC), with study details, basic patient clinicopathological characteristics, RS data, LRR rates, surgical data, and adjuvant treatment strategies all recorded. The final search was performed on the 22nd October 2021.

### Data management and analysis

Descriptive statistics were used to outline characteristics of included studies (Fisher’s Exact (†) and Chi-Squared (χ^2^) tests as appropriate). Rates of LRR for each RS group were expressed as dichotomous or binary outcomes, reported as odds ratios (ORs) and risk ratios (RRs) were expressed with 95% confidence intervals (CIs). ORs/RRs were calculated, using crude event study data, to compare interventions using per-protocol data, where applicable. The lowest RS cut-off category (traditional: RS < 18, TAILORx: RS < 11) were used as the principal comparator for all analyses.

Frequentist NMAs were conducted using netameta and Shiny packages for R [14]. LRR effect were described with a 95% CI. Results were considered statistically significant at the *P* < 0.050 level if the 95% CI did not include the value of one. Rank probabilities were plotted against the possible ranks for all competing treatments. As included studies were non-randomized, observational studies, methodological assessment was undertaken using the Newcastle–Ottawa Scale [15].

## Results

### Literature search and study characteristics

**Fig. 1:**
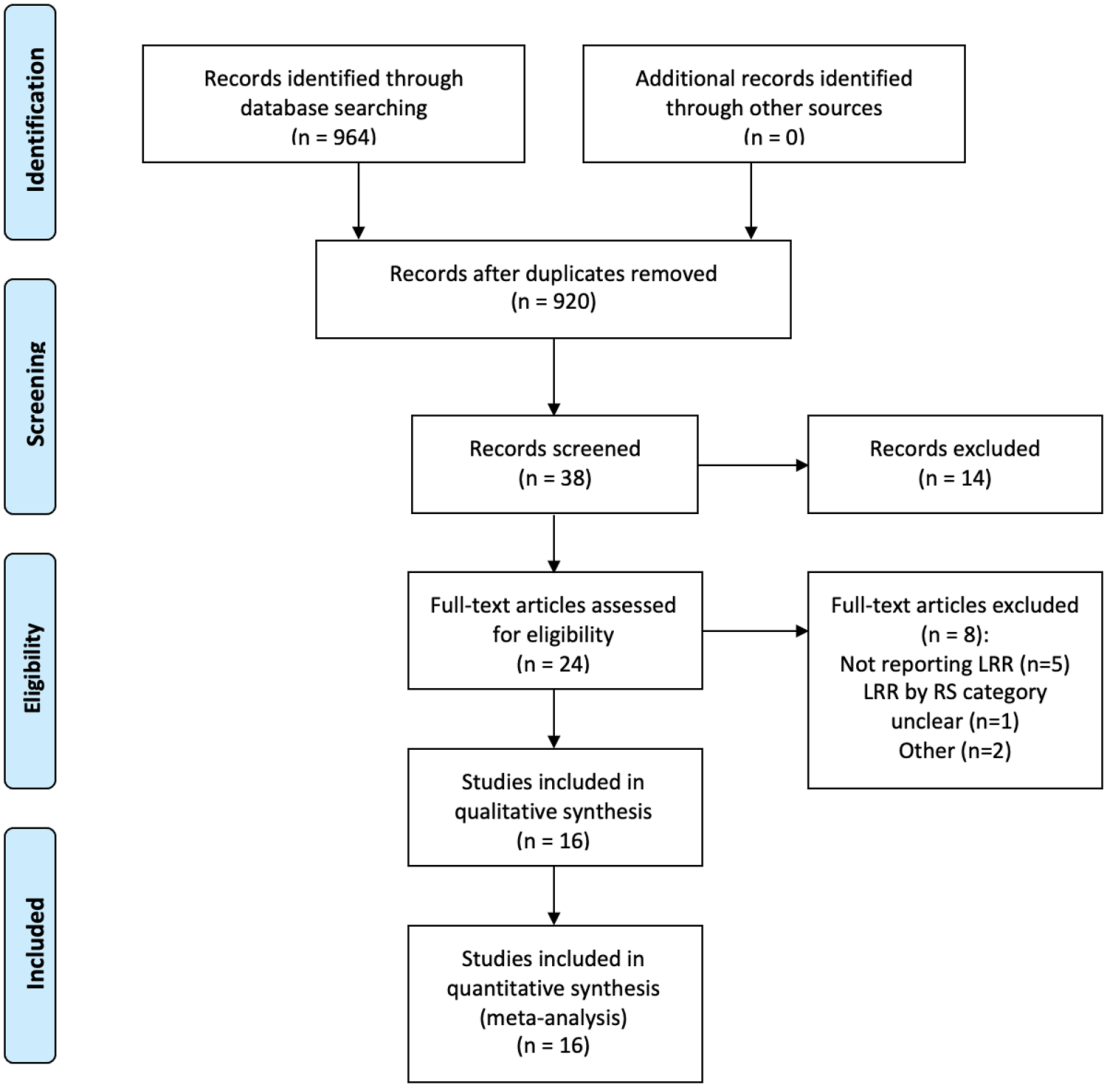
PRISMA flowchart outlining the systematic search process

The systematic search strategy identified a total of 964 studies, of which 44 duplicate studies were manually removed. The remaining 920 titles were screened for relevance, before 89 abstracts were reviewed. In total, we evaluated 24 full-text manuscripts and 16 studies fulfilled our inclusion criteria and were included in this systematic review and NMA [8, 16–30] (Fig. 1). Of the 16 studies included in this analysis, 56.3% were conducted in research institutions in the United States of America (9/16). In total, 37.5% of the included studies were prospective in design (6/16) and publication dates ranged from 2010 to 2021 (Table 1).

**Table 1:** Table summarizing the included studies in this systematic review and network meta-analysis

Author	Year	Country	Level of evidence	Follow up (months)	Number	Age in years (range)	NOS	Definition of LRR (if provided)
Abdelhakam	2021	Egypt	Retrospective	–	100	62.4 (43–85)	6	–
Davey	2021	Ireland	Retrospective	74.1	400	56.2 (27–75)	7	–
Jaafar	2014	UAE	Retrospective	31.2	47	48.0 (25–71)	6	–
Kim	2020	Korea	Retrospective	62.0	339	47.0 (29–77)	7	Recurrences in the ipsilateral breast, axillary, supraclavicular, and/or internal mammary nodes
Koh	2021	Korea	Retrospective	58.5	446	48.0 (27–77)	7	Development of DCIS or invasive cancer in the ipsilateral breast parenchyma, chest wall, axillary, internal mammary, or supraclavicular nodes
Lu	2021	China	Retrospective	61.5	1287	58.0 (24–91)	7	Recurrence in the chest wall, ipsilateral breast, or regional nodes
Mamousas	2010	USA	Prospective	120.0	1674	–	8	Ipsilateral breast tumor recurrence, chest wall recurrence, and regional nodal recurrence
Mamousas	2017	USA	Prospective	120.0	1065	–	8	–
Schwartzberg	2018	USA	Prospective	55.0	114	63.8 (44–96)	7	–
Solin	2012	USA	RCT	116.4	187	–	9	Recurrence in the treated breast and/or ipsilateral regional lymph nodes as the first site(s) of recurrence, with or without simultaneous distant metastases
Sparano	2018	USA	RCT	96.0	9719	56.0 (23–75)	9	–
Tevis	2019	USA	Retrospective	23.3	1121	56.0 (27–86)	7	–
Turashvilli	2017	USA	Retrospective	53.0	2326	57.0 (22–90)	7	Invasive breast cancer involving the ipsilateral breast parenchyma, axilla, regional lymph nodes, chest wall, or skin identified more than six months from the initial diagnosis of breast cancer
Woodward	2020	USA	RCT	98.4	316	60.4 (44–81)	9	Recurrence in the breast, chest wall, or axillary, infraclavicular, supraclavicular, or internal mammary lymph nodes
Yang	2019	Taiwan	Retrospective	36.8	138	–	6	–
Yang	2020	USA	Retrospective	29.0	1758	–	8	Ipsilateral breast/chest wall or regional nodal recurrence

*UAE* The United Arab Emirates, *USA* The United States of America, *RCT* randomized controlled trial, *NOS* Newcastle–Ottawa Scale, *DCIS* ductal carcinoma in situ

### Clinical characteristics

Overall, there were data included from 21,037 patients with mean age at diagnosis of 55.1 years (range: 22–96 years). The mean RS was 17.1 (range: 0–71) and the mean follow-up was 66.4 months (range: 27.0–120.0 months). In total, 2.8% of included patients experienced LRR (590/21,037).

### Treatment characteristics

In total, 64.0% of patients underwent breast-conserving surgery (BCS) (12,060/19,130–12 studies) and 33.1% underwent mastectomy (6277/18,943–11 studies). Of those reporting treatment with adjuvant radiotherapy (XRT), 99.2% of patients eligible for BCS received breast XRT (5297/5339–11 studies). Following RS testing, 43.5% of patients received combined chemoendocrine therapy (9142/21,037–16 studies). The majority of patients underwent endocrine therapies (90.5%, 19,028/21,037–16 studies).

### Locoregional recurrence rates: Traditional cut-offs

When using traditional RS cut-offs, 49.7% of patients had tumors with RS < 18 (3944/7935), 33.8% had RS 18–30 (2680/7935), and 16.5% had RS > 30 (1311/7935). Alternatively, 52.0% of patients had RS > 18 (4269/8213) and 83.5% of patients had RS < 30 (6610/7921) (Table 2). LRR rates increased in a stepwise fashion in accordance with traditional RS categories: RS < 18: 2.2% vs. RS 18–30: 3.5% vs. RS > 30: 10.2% (*P *< 0.001, χ^2^). Additionally, LRR rates increased when comparing dichotomous RS cut-offs: RS < 18: 2.2% vs. RS > 18: 6.0% (*P* < 0.001, †) and RS < 30: 2.7% vs. RS > 30: 10.2% (*P* < 0.001, †) (Table 2).

**Table 2:** Locoregional recurrence rates per 21-gene recurrence score category

Traditional Cut-Offs					
Author	RS < 18	RS > 18	RS18-30	RS < 30	RS > 30
Abdelhakam	2/57	0/43	0/39	2/96	0/4
Jaafar	0/25	0/22	0/19	0/44	0/3
Lu	1/361	26/926	7/685	8/1046	19/241
Mamousas	47/862	93/812	33/368	80/1230	60/444
Mamousas	13/386	64/769	26/364	39/750	38/315
Schwartzberg	–	–	–	2/107	0/7
Solin	3/82	10/105	4/63	7/145	6/42
Tevis	2/656	3/465	1/365	3/1021	2/100
Turashvilli	13/1394	31/932	22/777	35/2171	9/155
Woodward	7/121	27/195	–	–	–
Total	88/3944	254/4269	93/2680	176/6610	134/1311

TAILORx Cut-Offs					
Author	RS < 11	RS > 11	RS11–25	RS < 25	RS > 25
Davey	1/46	5/354	4/294	5/340	1/60
Kim	0/55	4/284	0/241	0/296	4/43
Koh	–	–	–	9/277	12/169
Schwartzberg	1/25	1/89	1/72	2/97	0/17
Sparano	20/1619	177/8100	139/6711	159/8330	38/1389
Tevis	1/229	4/892	2/718	3/947	2/174
Yang	–	–	–	3/121	2/17
Yang	–	–	–	18/1429	4/329
Total	23/1974	191/9719	146/8036	199/11,837	63/2198

**Fig. 2:**
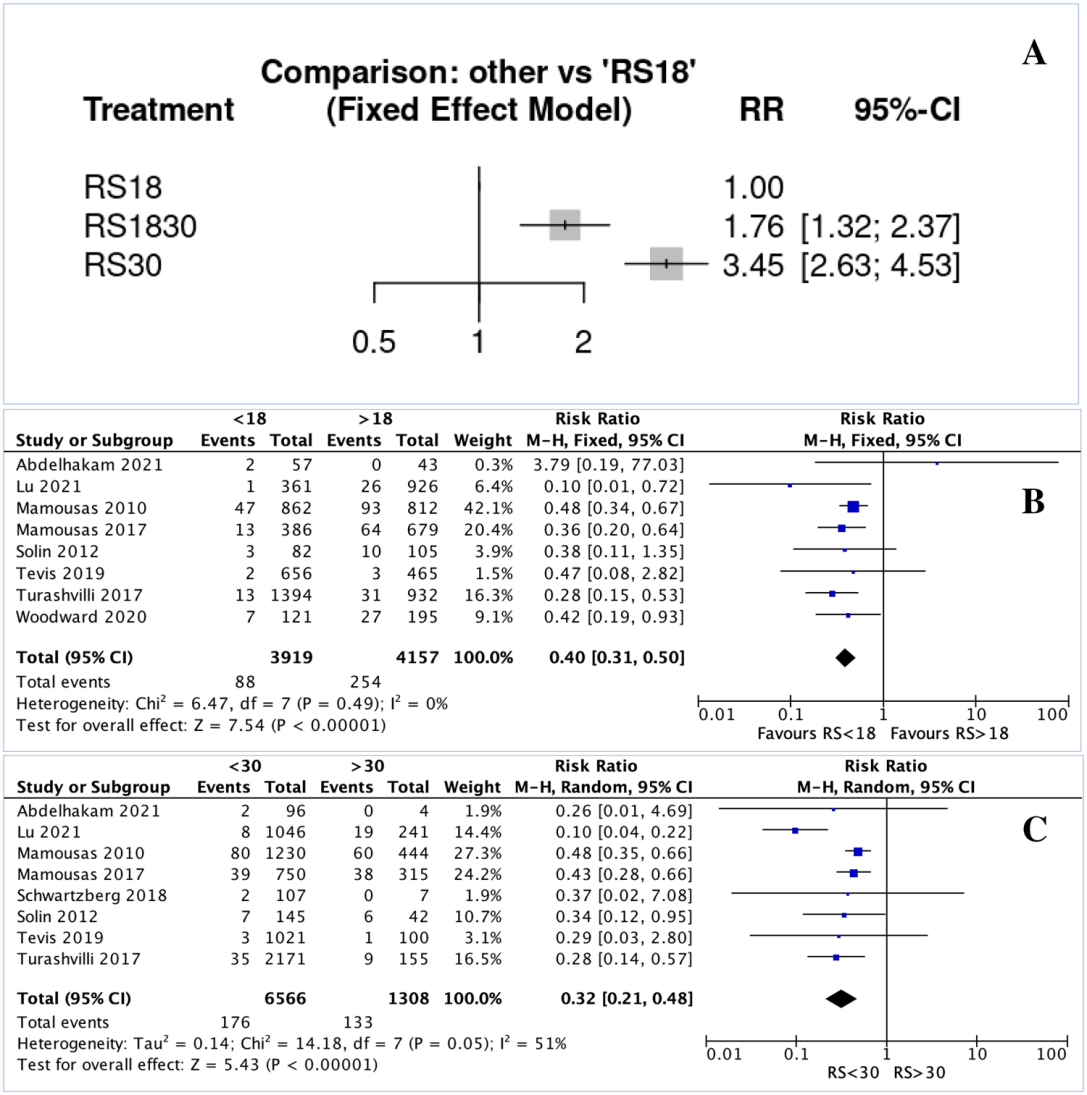
Forest plots illustrating the locoregional recurrence per 21-gene recurrence score expression assay group: **A** Network plot estimating the risk of locoregional recurrence for patients with RS 18–30 and RS > 30 versus RS < 18; **B** forest plot estimating the risk of locoregional recurrence for patients with RS < 18 versus those with RS > 18; and **C** forest plot estimating the risk of locoregional recurrence for patients with RS < 30 versus those with RS > 30

In the NMA, patients with RS 18–30 (RR 1.76, 95% CI 1.32–2.37) and RS > 30 (RR 3.45, 95% CI 2.63–4.53) were significantly more likely to experience LRR than those with RS < 18 (Fig. 2.A). Patients with RS < 18 were significantly less likely to experience LRR versus those with RS ≥ 18 (RR 0.40, 95% CI 0.31–0.50, *P* < 0.001, I^2^ = 0%) (Fig. 2.B). Patients with RS < 30 were significantly less likely to experience LRR versus those with RS ≥ 30 (RR 0.32, 95% CI 0.21–0.48, *P* < 0.001, I^2^ = 51%) (Fig. 2.C). Forest plots illustrating LRR risk based on nodal status using traditional RS cut-offs are illustrated in Supplementary Appendices 1.A–1.B.

### Locoregional recurrence rates: TAILORx cut-offs

Using TAILORx cut-offs, 16.2% of patients had tumors with RS < 11 (1974/12,208), 65.8% had RS 11–25 (8036/12,208), and 18.0% with RS > 30 (2198/12,208). Alternatively, 83.1% of patients had tumors with RS > 11 (9719/11,693) and 84.3% had RS < 30 (11,837/14,035) (Table 2). Once again, LRR rates increased in a stepwise fashion in accordance with traditional RS categories: RS < 11: 1.2% vs. RS 11–25: 1.8%, vs. RS > 25: 2.9% (*P* < 0.001, χ^2^). LRR rates increased using dichotomous cut-offs: RS < 11: 1.2% vs. RS ≥ 11: 2.0% (*P* = 0.016, †) and RS < 25: 1.7% vs. RS ≥ 25: 2.9% (*P* < 0.001, †) (Table 2).

**Fig. 3:**
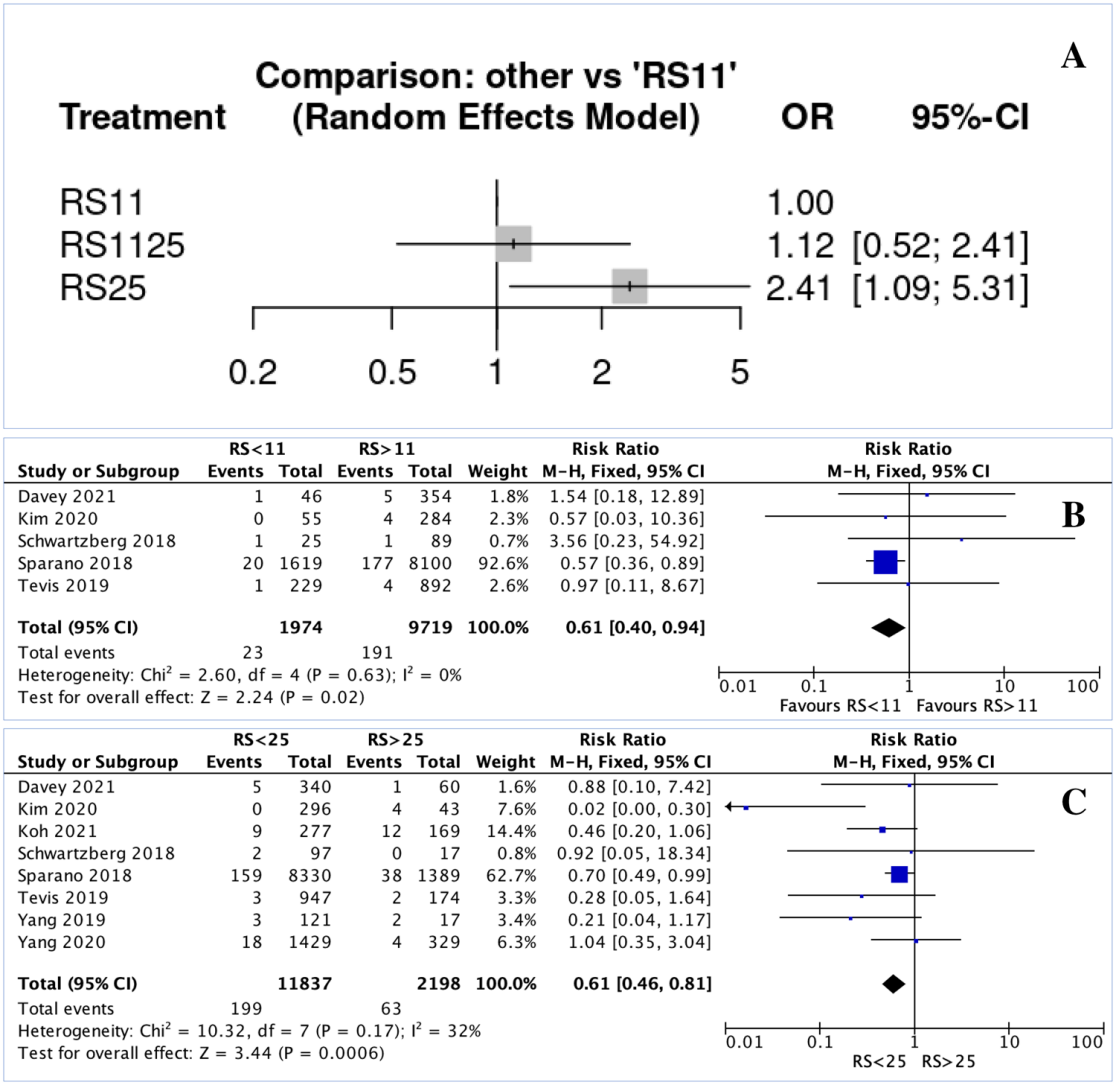
Forest plots illustrating the locoregional recurrence per 21-gene recurrence score expression assay group: **A** Network plot estimating the risk of locoregional recurrence for patients with RS 11–25 and RS > 25 versus RS < 11; **B** forest plot estimating the risk of locoregional recurrence for patients with RS < 11 versus those with RS > 11; and **C** forest plot estimating the risk of locoregional recurrence for patients with RS < 25 versus those with RS > 25

In the NMA, there were similar LRR rates for patients with RS 11–25 (RR 1.120, 95% CI 0.510–2.410) and RS > 25 (RR 2.410, 95% CI 1.090–5.310) compared to those with RS < 11 (Fig. 3.A). Patients with RS < 11 were significantly less likely to experience LRR than those with RS > 11 (RR 0.610, 95% CI 0.400–0.940, *P *= 0.020, I^2^ = 0%) (Fig. 3.B). Patients with RS < 25 were significantly less likely to experience LRR versus those with RS > 25 (RR 0.610, 95% CI 0.460–0.810, *P* < 0.001, I^2^ = 32%) (Fig. 3.C). Forest plots illustrating LRR risk based on nodal status using TAILORx RS cut-offs are illustrated in Supplementary Appendices 1.A–1.B.

## Discussion

To our knowledge, this is the first systematic review evaluating patient risk of LRR following the substratification and treatment of early-stage ER+/HER2− breast cancer in accordance with the 21-gene RS expression assay. The results of this study illustrate a stepwise increase in LRR rates with increasing RS, regardless of the use of traditional or TAILORx cut-offs. The clinical application of RS testing has transformed the management paradigm of early-stage ER+/HER2− disease and has facilitated the personalization of combined chemoendocrine therapy for those at the greatest risk of distant disease recurrence [5], while minimizing probable overtreatment for those who will benefit little from such therapies [8, 9]. While the 21-gene assay expression assay testing has been focused on controlling distant disease recurrence, the results of this study illustrate a significant correlation between LRR rates and RS category. Therefore, the current study highlights the role for biomolecular and genomic tumor features in providing valuable information in relation to LRR risk in ER + disease. This data may be useful in facilitating the appropriate de-escalation and/or escalation of adjuvant treatment strategies being utilized to establish locoregional control of the breast and axilla.

In this systematic review, we observed LRR rates of 2.2%, 3.5%, and 10.2% when applying traditional cut-offs with respect to low-, intermediate-, and high risk for patients (*P* < 0.001, χ^2^). While these findings are similar to the LRR rates observed in the seminal NSABP-B14 and B-20 trials at 10-year follow-up (LRR rates of 4.3%, 7.2%, and 15.8%, respectively), this comparison is limited by the shorter duration of mean follow-up in the current analysis (5 and a half years vs. 10-year follow-up in NSABP-B14/B-20). Additionally, we acknowledge this is not a matched comparison due to our inclusion of LN + disease in the current study. Of note, the data outlined in the study by Mamousas et al. contribute a large proportion to the LRR rates observed in each of the RS groups delineated using traditional cut-offs, which is best explained through their inclusion of node-positive cancers only (based on the inclusion criteria of NSABP B28) [23]. In our NMA, the relative risk of LRR was 1.76 times more likely in patients with RS 18–30 and 3.45 times more likely in those with RS > 30, compared to those with RS < 18. The crude data in this study illustrate a significant difference in LRR rates when using RS 18 (RS < 18: 2.2% vs. RS ≥ 18: 6.0%, *P* < 0.001, †) and RS 30 (RS < 30: 2.7% vs. RS ≥ 30: 10.2%, *P* < 0.001, †) as cut-offs to delineate the risk of LRR and those with lower RS categories were significantly less likely to experience LRR at meta-analysis (RS < 18: RR 0.40, RS < 30: RR 0.32). These are interesting findings that the commercially available 21-gene expression assay has been only validated to quantify the risk of distant disease recurrence in those diagnosed with ER+/HER2− breast cancer, by successfully selecting those with tumors of more aggressive biology to receive combined chemoendocrine therapy. Based on the results of this analysis, the RS has potential clinical utility in estimating safe locoregional control and risk of LRR, hereby challenging recommendations made at the 17th St. Gallen expert consensus. The 2021 panel recommended that genomic testing should not be utilized in guiding adjuvant regional node irradiation (92% against) and post-mastectomy radiotherapy (PMRT) (89% against) in patients with ER + disease [31]. While traditional parameters such as clinicopathological and surgical data (i.e., tumor staging, and margin status) will remain important in guiding therapeutic decision-making in relation to XRT to aid locoregional control following tumor resection, the present study supports the rationale that patients with a RS < 18 have a very low incidence of LRR. This may have implications for therapeutic decision-making and the judicious use of RS to aid therapeutic adjuvant decision-making in relation to XRT in future clinical trials.

When applying the TAILORx cut-offs, this stepwise increase in LRR rates remained consistent [RS < 11: 1.2%, vs. RS 11–25: 1.8%, vs. RS > 25: 2.9% (*P* < 0.001, χ^2^)] and increased again when applying dichotomous cut-offs [RS < 11: 1.2% vs. RS ≥ 11: 2.0% (P = 0.016, †), RS < 25: 1.7% vs. RS ≥ 25: 2.9% (*P* < 0.001, †)]. In our NMA, the relative risk of LRR was similar for RS < 11 versus RS 11–25, however was 2.41 times more likely in those with RS > 30. Furthermore, those with lower RS were significantly less likely to experience LRR at meta-analysis (RS < 11: RR 0.61, RS < 25: RR 0.61). However, caution must be taken when interpreting these results. The inclusion of data from the prospective TAILORx trial represents a large proportion of the patients evaluated using TAILORx cut-offs (69.3%, 9719/14,035), which inevitably heavily influences LRR outcomes for this cohort [8]. While these data from Sparano et al. may be considered to ‘skew’ results and analyses, we must acknowledge the inherent value of this data; TAILORx was a prospective, multicenter trial of randomized design, indicating reliable evidence which accurately indicates the risks of LRR for these patients. Therefore, relying on this study to provide ‘real-world’ risk of LRR by RS category is justifiable, particularly when the capabilities of RS in predicting LRR in early-stage ER+/HER2− breast cancer is likely being underestimated (and subsequently underutilized) in establishing LRR in high-risk cases.

The current NMA outlines the risk of LRR based on results of the 21-gene expression assay in ER+/HER2− disease. As outlined by Goldhirsch et al. at the 2013 St. Gallen International Expert Consensus [32], genomic substratification classifies high-risk ER+/HER2− cases (such as those with RS > 30: RR 3.45 and RS > 25: 2.41) replicate the more aggressive luminal B molecular subtype. In their meta-analysis of over 12,000 patients, Lowery et al. outlined the importance of steroid hormone receptor and HER2 status in establishing the risk of LRR, with those with ER+/HER2− cancers being considered to have a lower relative risk of LRR compared to triple-negative (OR: 0.38) and HER2-positive (OR: 0.34) molecular phenotypes [33]. However, this analysis failed to establish the LRR rates for luminal A and B diseases independently. In their later systematic review, McGuire et al. established there is reduced risk of LRR among patient with luminal A disease compared to all other molecular subtypes [34]. Interestingly, McGuire et al. observed a LRR rate of 1.7% for patients with luminal A disease after 53-month follow-up, with LRR rates of 3.3% in those with luminal B disease (albeit limited in that they included HER2+cancers). These results are similar to the observed 2.8% of patients experiencing LRR (590/21,037) after 66.4-month follow-up in the current study, with LRR rates of 1.7% for RS < 25 and 2.9% for RS ≥ 25 (*P* < 0.001, †). While these previous studies evaluated LRR risk by receptor status, this NMA has the advantage of solely quantifying LRR risk using the 21-gene expression assay in ER+/HER2− disease, highlighting the value of genomic testing in estimating LRR rates in this subgroup of breast carcinoma.

The present study has several limitations. Firstly, and most importantly, the studies failed to provide data to allow accurate subgroup analysis in relation to LRR adjusted for age, menopause status, margin positivity levels, chemotherapy prescription, and most importantly, surgical management and XRT use. Therefore, conclusions drawn as to how to best apply RS testing to influence locoregional control of the breast and axilla may be perceived to be somewhat limited. Secondly, data from the prospective TAILORx trial represent a large proportion of the patients evaluated in this NMA using TAILORx cut-offs (69.3%), which heavily influences these outcomes in relation to LRR. Similarly, the patients included in this study by Sparano et al. represent 46.2% of the total patients included (9719/21,037). Thirdly, just 37.5% of the included studies were of a prospective design (6/16), indicating most studies were retrospective, observational studies which are inherently subjected to ascertainment, confounding, and selection biases. Finally, publication dates of included studies ranged from 2010 to 2021, during which period the seminal results of TAILORx and RxPONDER have transformed the management paradigm for patients diagnosed with early-stage ER+/HER2− breast cancers. Despite these limitations, the current study is the first systematic review to provide real-world data estimating the risk of LRR based on results of the results of genomic testing for early-stage breast carcinoma. Moreover, this analysis contributes to current efforts focused on expanding indications for the 21-gene expression assay in the modern breast cancer treatment paradigm [35–38].

In conclusion, RS testing accurately estimates the risk of LRR for patients being treated with curative intent for early-stage ER+/HER2− breast cancer. While RS testing is validated for quantifying the risk of distant disease recurrence, awareness for its ability to predict LRR is essential to establish effective locoregional control of the breast and axilla. Future prospective, randomized studies may further validate the predictive value of RS in estimating LRR and the application of RS to establish adequate locoregional control in high-risk cases.

## Supplementary Information

Below is the link to the electronic supplementary material.

Supplementary file1 (DOCX 303 KB)

## Data Availability

Study data will be made available upon reasonable request from the corresponding author.
