# Supplementary material for: Value of the 21-gene expression assay in predicting locoregional recurrence rates in estrogen receptor-positive breast cancer: a systematic review and network meta-analysis
Source: Breast Cancer Res Treat. 2022 Apr 15;193(3):535–44. doi: 10.1007/s10549-022-06580-w (PMC9114034; doi:10.1007/s10549-022-06580-w)
Supplement: Supplementary file 1 — Supplementary file1 (DOCX 303 KB) [file 10549_2022_6580_MOESM1_ESM.docx]

**Value of the 21-Gene Expression Assay in Predicting Locoregional Recurrence Rates in Estrogen Receptor Positive Breast Cancer: A Systematic Review and Network Meta-Analysis**

Matthew G. Davey MCh MRCS, Eoin F. Cleere MB BCh, John P. O’Donnell MCh FRCS, Sara Gaisor, Aoife J. Lowery PhD FRCS, Michael J. Kerin, MCh FRCSI

Department of Surgery, The Lambe Institute for Translational Research, National University of Ireland, Galway, Galway H91 YR71, Republic of Ireland.

**SUPPLEMENTARY APPENDICES**


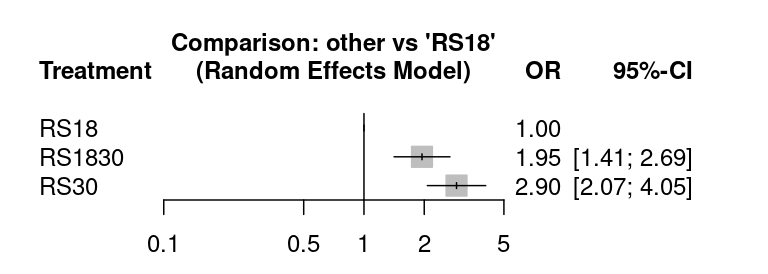


A

B


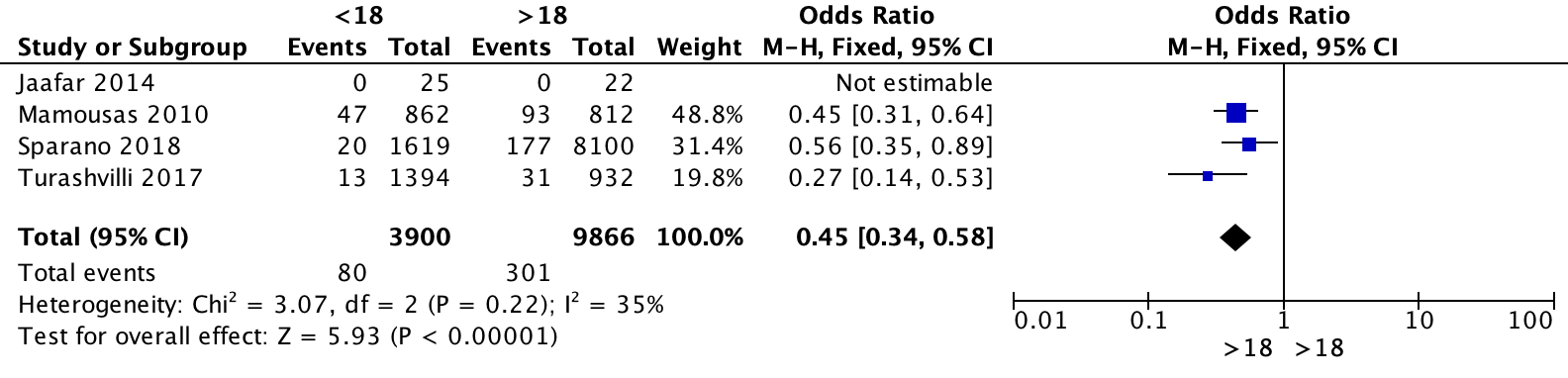


C


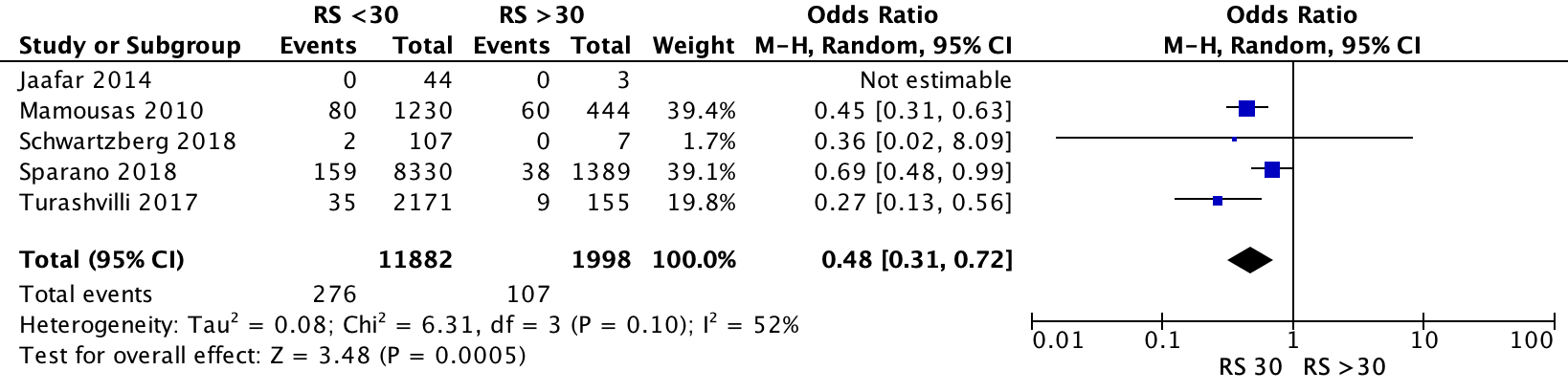


**Supplementary Appendix 1A.**

Forest plots illustrating the locoregional recurrence per 21-gene recurrence score expression assay group for those with lymph node negative disease using the traditional cut-offs:

(A) Network plot estimating the risk of locoregional recurrence for patients with RS 18-30 and RS>30 versus RS<18; (B) Forest plot estimating the risk of locoregional recurrence for patients with RS<18 versus those with RS>18; and (C) Forest plot estimating the risk of locoregional recurrence for patients with RS<30 versus those with RS>30.


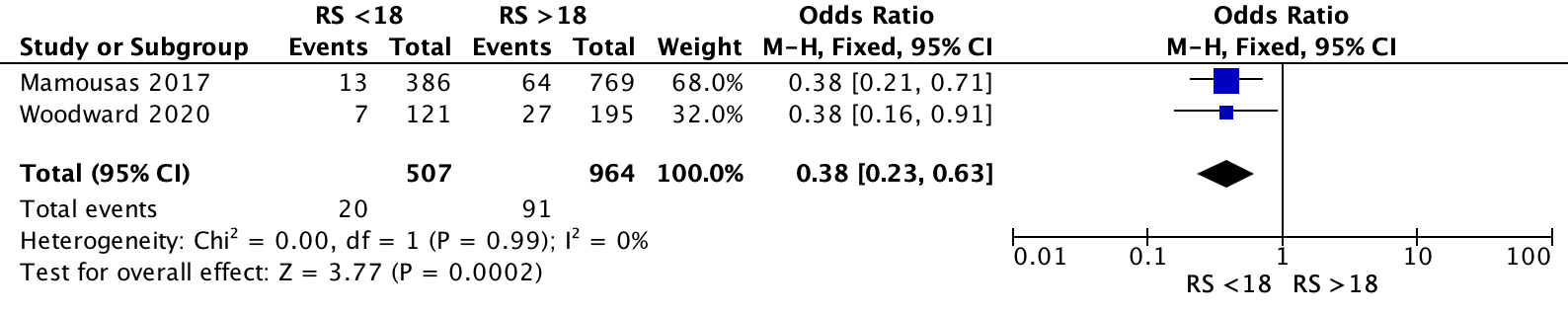


**Supplementary Appendix 1B.**

Forest plot illustrating the locoregional recurrence per 21-gene recurrence score expression assay group (RS<18 versus those with RS>18) for those with lymph node positive disease.


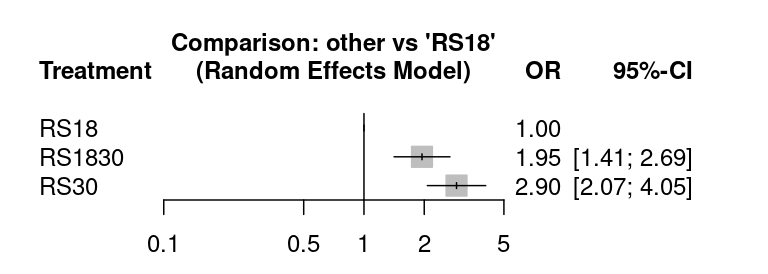


A

B


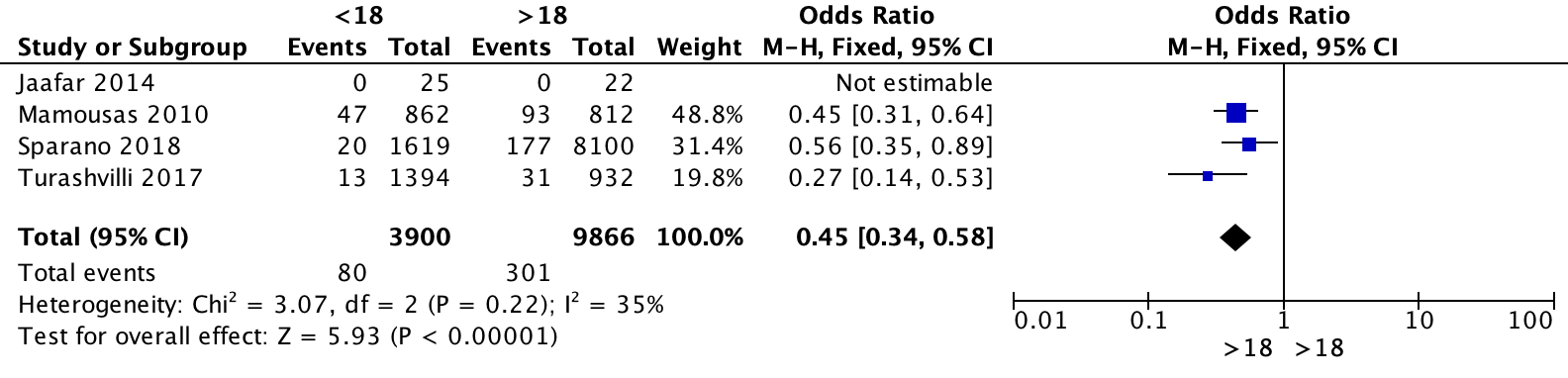


C


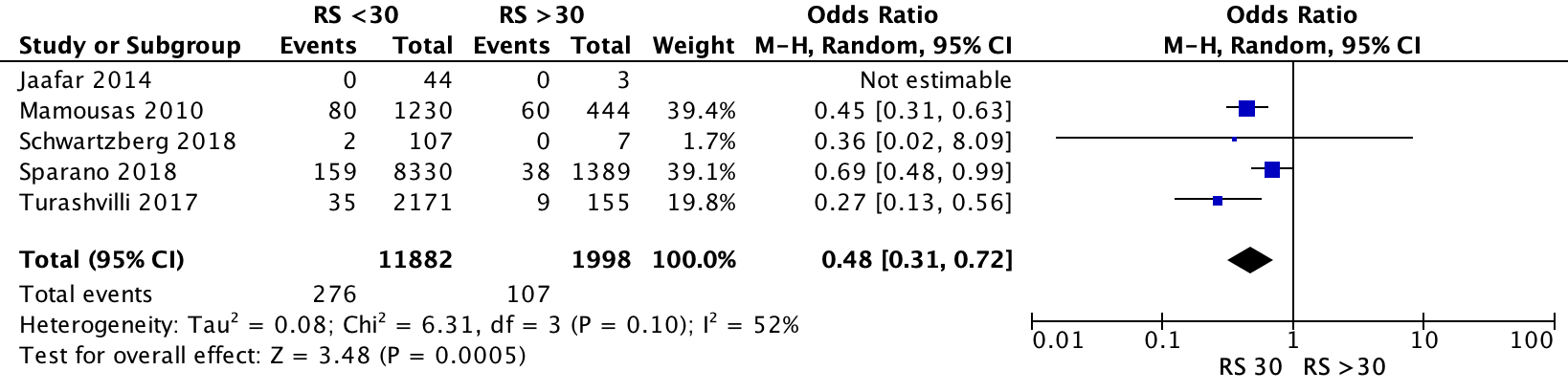


**Supplementary Appendix 1C.**

Forest plots illustrating the locoregional recurrence per 21-gene recurrence score expression assay group for those with lymph node negative disease using the traditional cut-offs:

(A) Network plot estimating the risk of locoregional recurrence for patients with RS 18-30 and RS>30 versus RS<18; (B) Forest plot estimating the risk of locoregional recurrence for patients with RS<18 versus those with RS>18; and (C) Forest plot estimating the risk of locoregional recurrence for patients with RS<30 versus those with RS>30.
